# A neurosurgical approach to traumatic brain injury and post-traumatic hypopituitarism

**DOI:** 10.1007/s11102-018-0925-z

**Published:** 2018-11-27

**Authors:** Chin Lik Tan, Peter J. Hutchinson

**Affiliations:** 1 Division of Neurosurgery, National University Hospital, 5 Lower Kent Ridge Road, Singapore, 119074 Singapore; 2 Division of Neurosurgery, Department of Clinical Neurosciences, University of Cambridge, Cambridge Biomedical Campus, Box 167, CB2 0QQ Cambridge, UK

**Keywords:** Traumatic brain injury, Hypopituitarism, Neurosurgery

## Abstract

**Purpose:**

Traumatic brain injury (TBI) is a common cause of mortality and major disability worldwide. The initial management often depends on the severity of the injury. Pituitary dysfunction can develop as a sequela of TBI, and can have long-term, debilitating impact on the patients. Early identification and prompt intervention of post-traumatic hypopituitarism (PTHP) is essential to prevent or minimize the adverse consequences of this condition. We hereby provide an overview of the current management of TBI from a neurosurgical standpoint. We then review the pathophysiology and risk factors of developing PTHP, as well as our recommendations for its management.

**Methods:**

A review of current literature on TBI and PTHP, including primary research articles, reviews and clinical guidelines.

**Results:**

The current neurosurgical approach to the management of TBI is presented, followed by the pathophysiology and risk factors of PTHP, as well as our recommendations for its management.

**Conclusions:**

Post-traumatic hypopitutiarism is a serious and potentially debilitating condition that is likely under-recognised and under-diagnosed. From a neurosurgical perspective, we advocate a pragmatic approach, i.e. screening those considered at high risk of developing PTHP based on clinical features and biochemical/endocrinological testings; and referring them to a specialist endocrinologist for further management as indicated.

## Traumatic brain injury

Traumatic brain injury (TBI) is a serious, common and potentially devastating condition which is a major cause of mortality and major disability in the developed and developing world alike. Worldwide, it is estimated that approximately 69 million people suffer from TBI each year, with Southeast Asia and Western Pacific regions experiencing the highest burden of disease [1]. In the developed world, North America and Europe have an estimated incidence of 1,299/100,000 and 1,012/100,000 per year, respectively [1].

The leading causes of TBI are falls, road traffic accidents and assault. Males are twice as likely to suffer from TBI as females. With an increasingly aging population, the incidence of TBI among the elderly is also increasing, with falls being the major contributor. In the United States, more than 80,000 attendances to the Emergency Department per year were made by patients aged 65 or older, three-quarter of whom require hospitalization [2].

TBI can be classified according to the post-resuscitation Glasgow Coma Scale (GCS) score, into mild (GCS 13–15), moderate (GCS 9–12) and severe (GCS 3–8). Patients who sustained severe TBI often require hospitalization ranging from weeks to months. Many of these patients end up in coma or becoming fully dependent on others for round-the-clock care. Even among those with mild or moderate TBI, long-term sequelae may entail, resulting in chronic headache, dizziness, attention or cognitive deficits, memory problems and behavioural issues. Some of these symptoms may be identified as part of post-concussion syndrome (PCS), but others may also suffer from pituitary dysfunction or chronic traumatic encephalopathy. These complications, if untreated, may lead to impaired functional status, loss of productivity and poor quality of life.

## Management of traumatic brain injury

Following a traumatic insult, the brain suffers an immediate injury as a result of the mechanical impact. This is termed primary injury and is irreversible. However, this may trigger a range of physical, biochemical and cerebrovascular changes that can result in further injuries, i.e. secondary injury to the brain. This includes cerebral ischaemia, oedema, disturbed metabolism and excitotoxicity, and are reversible if managed correctly and promptly. However, if untreated, they can have long term deleterious outcome on the patient. The main goal of acute neurosurgical care of TBI patients is the prevention of secondary injuries.

The initial management of TBI patients depends primarily on the severity of the injury as assessed by the GCS score, taking into consideration other factors such as associated injuries (in a polytrauma setting) and pre-morbid status.

Severe TBI patients are managed according to the Brain Trauma Foundation Guidelines for the Management of Severe Traumatic Brain Injury [3]. The primary aim of management of severe TBI patients is the prevention and treatment of secondary brain injury, in order to preserve maximal amount of normally-functioning brain tissue. Patients with severe TBI should be transferred to a neurosurgical centre with expertise in neurotrauma care. This enables management in an intensive care setting, with intensive monitoring and correction of basic physiological parameters (e.g. blood pressure, pulse rate, oxygen saturation, temperature etc.), metabolic derangements (e.g. electrolytes, glucose, nutritional status etc.), neurological status (GCS score, pupil size and reactivity, focal neurological deficits etc.) and intracranial pressure (ICP). The most basic target is to regulate/lower the ICP and optimize cerebral perfusion pressure (CPP). This is initially done using medical measures, such as sedation, osmotherapy (mannitol or hypertonic saline), hyperventilation, maintenance of optimal blood pressure, oxygenation and temperature, as well as prevention and treatment of seizures and infection. An external ventricular drain (EVD) may also be inserted into the ventricular system to drain the cerebrospinal fluid (CSF) to lower the ICP. However, if the ICP remains refractory to medical treatment, surgery in the form of decompressive craniectomy may be indicated.

Patients who have sustained mild TBI are managed quite differently. Upon attendance to the Emergency Department, a set of criteria based on patient’s clinical conditions may be used to guide the necessity for performing a CT head scan [4]. Depending on the patient’s GCS score, neurological status, CT scan findings, as well as other symptoms or signs and co-morbidities, the patient may either be admitted for further monitoring or discharged from the Emergency Department. Those admitted will be monitored for GCS score and focal neurological deficits; if patients remain stable during this period of monitoring, they will be typically discharged after 24–48 h.

## Post-traumatic hypopituitarism

Post-traumatic hypopituitarism (PTHP) was first described nearly a century ago in 1918 [5]. However, it did not gain much attention until the recent two decades or so. It is now accepted that PTHP is a poorly-recognised and under-diagnosed sequela following TBI. Nevertheless the true prevalence of PTHP remains a matter of controversy, with studies reporting widely disparate figures, to as high as almost 70%. These inconsistencies may be attributed to the differences in inclusion/exclusion criteria, tests employed (e.g. dynamic vs static tests), timings of the testing (acute vs chronic phases) and threshold for diagnosis [6, 7]. Our recent review article includes a compilation of studies looking at the prevalence of abnormal endocrine tests in patients with TBI [8].

Pituitary dysfunction can lead to physical, psychological and cognitive complications. Acute pituitary dysfunction can result in adrenal insufficiency or diabetes insipidus (DI) which can cause haemodynamic instability and derangements in fluid balance. In the longer term, PTHP patients could suffer from attention and cognitive deficits, impaired quality of life and poor rehabilitation outcome [9–11]. Many neuropsychiatric manifestations following TBI including depression, anxiety, loss of emotional well-being have also been linked to PTHP, and have been documented up to 30% of patients [12]. Hence it is crucial to identify PTHP, both in the acute as well as chronic phases, in order to institute appropriate and timely treatment.

## Pathophsiology of post-traumatic hypopituitarism

Various theories have been proposed as possible pathophysiological mechanisms underlying PTHP. The most widely-accepted theory is that of an ischaemic insult to the pituitary gland [13, 14]. TBI can cause direct trauma to the pituitary gland [15, 16], transection/disruption of the pituitary stalk [17–19] or direct damage to the hypophyseal portal veins [17, 20]. Additional insults in the form of hypotension, hypoxia and brain swelling often seen in TBI may further exacerbate the injury, resulting in pituitary ischaemia and infarction [21].

Another possible mechanism is a direct mechanical impact to the pituitary gland. Being located in the sella turcica, the pituitary gland is particularly susceptible to injuries that involve basal skull fractures. It is believed that the more prevalent GH and gonadotrophin deficiencies in PTHP are due to the more laterally-located somatotrophs and gonadotrophs [22, 23]. Shearing of white matter tracts connecting the pituitary gland may also be injured by acceleration/deceleration forces exerted during TBI.

The role of autoimmunity in the development of PTHP has also been studied. TBI patients were found to have higher levels of anti-pituitary antibodies (APAs) and anti-hypothalamic antibodies (AHAs) at 3 and 5 years post-injury, compared to controls [24, 25]. High levels of APAs and AHAs were also detected in boxers, who frequently sustained chronic, repetitive insults to the brain. Higher levels of AHAs among boxers were found to be associated with higher prevalence of PTHP (46.2%) compared to controls (10.4%) [24, 26].

Post-mortem studies also helped unveil the pathological process that may underlie PTHP. Features including capsular haemorrhage around the pituitary (59%), posterior lobe haemorrhage (31%), anterior lobe necrosis (22%) and stalk necrosis (3%) [20, 27]. A more recent study in fatal closed TBI patients found high prevalence of pituitary stalk rupture and pituitary gland haemorrhage (43.3%), and that this was associated with the presence of subdural haemorrhage [28]. Ischaemic/haemorrhagic lesions in the hypothalamus have also been detected in approximately 42% of patients, suggesting a possibility of hypothalamic involvement in PTHP [15].

The advent of imaging technology also allowed closer inspection of radiological changes in and around the pituitary gland following TBI. Maiya et al. studied the MRI scans of 41 moderate-severe TBI patients and found evidence of pituitary enlargement in the acute phase (< 7 days). Other focal lesions were also detected in 30% of the patients, and included haemorrhage/haemorrhagic infarction, a swollen gland with bulging superior margin, heterogeneous signal in the anterior lobe, and partial transection of the infundibular stalk [19]. On the other hand, a study on longer-term cohort of TBI patients (mean 17.4 years after trauma), revealed a loss of pituitary volume or an empty sella, abnormal enhancement, perfusion deficits and/or lack of the posterior pituitary signal to be the main imaging findings [29]. Studying a cohort of soldiers who sustained blast TBI, Baxter et al. discovered that those who had pituitary dysfunction displayed greater white matter tract damage as assessed by diffusion tensor imaging [30]. The prevalence of skull/facial fractures was also higher among the soldiers with pituitary dysfunction (50%) compared to those without (0%) [30].

## Risks factors of post-traumatic hypopituitarism

It is still unknown which group of patients are more likely to develop pituitary dysfunction following TBI. Various putative risks factors, covering clinical, radiological and genetic aspects, have been suggested from earlier studies.

A number of studies have identified the initial GCS score as a predictor for developing PTHP [31–35]. A meta-analysis of 14 studies found that patients with severe TBI (GCS < 9) had the highest rate of developing PTHP (35.5%). However, the authors also found that moderate TBI patients (GCS 9–12) had a lower rate of PTHP (10.9%) compared to mild TBI patients (GCS 13–15) (16.8%) [29]. However, findings from several other studies appeared to contradict this, suggesting instead that there exists no causal relationship between GCS score and PTHP [36–40].

Several groups also identified imaging findings that may be predictive of development of PTHP. This include findings from CT scan including diffuse brain swelling [31], basal skull fractures and diffuse axonal injury [39]. However, two studies did not find a correlation between PTHP and imaging findings of intracranial haematoma or diffuse axonal injury [32, 37].

Other factors associated with development of PTHP include old age [39], raised intracranial pressure [41, 42], hypotension and hypoxia [31], and prolonged ICU stay. Possession of apolipoprotein E3/E3 genotype was found to be associated with a lower risk of PTHP [43].

Mechanism of injury may also be associated with PTHP. Blast TBI has been found to confer higher risk of PTHP, based on studies on military personnel returning from warzones [30, 44]. Baxter et al. identified that patients who suffered moderate-severe blast TBI had a higher risk of developing PTHP (32.0%) compared to those with non-blast TBI (2.6%) [30]. Undurti et al., meanwhile, found high prevalence (31%) of PTHP among army veterans with prior blast mild TBI, which was associated with increased post-concussive symptoms [44].

## Recommendations for screening and management of post-traumatic hypopituitarism

A number of authors have put forward various recommendations for the management of PTHP [7, 45, 46]. While the details may differ, the general concepts appear similar.

On behalf of the British Neurotrauma Group, we recently published a set of guidance on the screening and management of pituitary dysfunction following TBI in adults [8]. The aim of the publication was two-fold: to provide guidance to clinicians (including neurosurgeons, intensive care specialists, surgeons and physicians) on who, when and how to screen for and manage pituitary dysfunction following TBI; and to provide a framework for future research in this area to further refine and update the recommendations as and when new evidence emerge [8].

### The acute phase in hospital

Various physiological changes occur in the acute phase following intercurrent illnesses, including TBI. This may affect levels of hormones detected, resulting in false positives. This makes interpretation of endocrine test results extremely challenging and potentially unreliable. Hence, in contrast to suggestions from other authors [7, 46], we do not recommend routine testing of endocrine function or assessment of plasma/serum cortisol level in the acute phase following TBI. However, if a patient displays signs of cortisol insufficiency (e.g. refractory hypotension, hypoglycaemia, hyponatraemia), cranial DI (e.g. hypernatraemia, hypotonic polyuria) or syndrome of inappropriate antidiuretic hormone secretion(SIADH) (e.g. euvolaemic hyponatraemia), appropriate investigations should be carried out and hormone replacement be given immediately [8].

### The post-discharge and chronic phase

Many authors recommend screening for patients who had moderate-severe TBI at 3–6 months to detect PTHP, using a combination of basal (non-stimulated), and where appropriate, dynamic tests [7, 47–50]. We recommend that all patients who are admitted for 48 h or more for TBI undergo screening for PTHP at 3–6 months. This should include renal function tests, thyroid function tests, cotisol level and sex hormone levels. If abnormality is detected, they should be referred to an endocrinologist to have a full assessment, including a dynamic testing for growth hormone deficiency (GHD) [8]. Patients who are not admitted, or who are admitted for less than 48 h should also undergo screening tests if they have on-going symptoms consistent with pituitary dysfunction. GHD is commonly transient, and does not require testing within 12 months. However, TBI patients who have persistent symptoms beyond 12 months should undergo testing for GHD and other pituitary abnormalities [8].

It must be borne in mind that many mild TBI patients may not be placed under the care of neurosurgeons. This is particularly true for elderly patients admitted for falls, who are often cared for by physicians throughout their hospital stay. Hence physicians who are frequently involved in the care of TBI patients (e.g. acute care physicians, geriatricians) should also be aware of the possibility of PTHP and follow these patients up accordingly.

## Conclusions

Post-traumatic hypopitutiarism is a serious and potentially debilitating condition that may afflict sufferers of TBI. While its true prevalence remains to be determined, it is likely that this condition is under-recognized and under-diagnosed. More concerted efforts are required to understand the epidemiology, pathophysiology, and risk factors in order to develop better screening tools and therapeutic strategies to improve the management of these patients.

Our current approach to PTHP can be considered a pragmatic one. The incidence of TBI (from mild to severe) is very high and it is impossible to screen everyone for pituitary dysfunction. Hence our strategy is to screen those considered at high risk of developing PTHP based on clinical features and biochemical/endocrinological testings; and when an abnormality is detected, to refer them to a specialist endocrinologist for further management. Nevertheless, we view this approach as a work in-progress, and should be updated and improved upon as and when new evidence becomes available.
